# Host Delivered RNAi, an efficient approach to increase rice resistance to sheath blight pathogen (*Rhizoctonia solani*)

**DOI:** 10.1038/s41598-017-07749-w

**Published:** 2017-08-08

**Authors:** Ila Mukul Tiwari, Arun Jesuraj, Richa Kamboj, B. N. Devanna, Jose R. Botella, T. R. Sharma

**Affiliations:** 1 National Research Centre on Plant Biotechnology, Pusa Campus, New Delhi 110012 India; 2 School of Agriculture and Food Sciences, The University of Queensland, Brisbane, QLD Australia; 3 National Agri-Food Biotechnology Institute (NABI), Mohali, Punjab India 160071

## Abstract

*Rhizoctonia solani*, the causal agent of rice sheath blight disease, causes significant losses worldwide as there are no cultivars providing absolute resistance to this fungal pathogen. We have used Host Delivered RNA Interference (HD-RNAi) technology to target two PATHOGENICITY MAP KINASE 1 (*PMK*1) homologues, *RPMK*1-1 and *RPMK*1-2, from *R. solani* using a hybrid RNAi construct. *PMK*1 homologues in other fungal pathogens are essential for the formation of appressorium, the fungal infection structures required for penetration of the plant cuticle, as well as invasive growth once inside the plant tissues and overall viability of the pathogen within the plant. Evaluation of transgenic rice lines revealed a significant decrease in fungal infection levels compared to non-transformed controls and the observed delay in disease symptoms was further confirmed through microscopic studies. Relative expression levels of the targeted genes, *RPMK*1-1 and *RPMK*1-2, were determined in *R. solani* infecting either transgenic or control lines with significantly lower levels observed in *R. solani* infecting transgenic lines carrying the HD-RNAi constructs. This is the first report demonstrating the effectiveness of HD-RNAi against sheath blight and offers new opportunities for durable control of the disease as it does not rely on resistance conferred by major resistance genes.

## Introduction

Rice is one of the most important cereal crops and a source of staple food for nearly half of the global population. More than 90% of rice is grown and consumed in Asia, where 55% of the world’s population resides. Rice sheath blight (ShB) caused by *Rhizoctonia solani* Kuhn, is one of the most destructive diseases worldwide and has been reported in all growing areas, including temperate, sub-tropical and tropical regions in diverse rice production systems, causing approximately 50% yield reduction in test plots of susceptible cultivars^1–4^. *R. solani* is an important soil-borne pathogen that attacks wide range of crops worldwide besides rice^5, 6^. The widespread adoption of high yield, agronomically superior rice cultivars with large numbers of tillers along with changes in cultural practices associated with these cultivars, favours the development of sheath blight and contributes to the rapid increase in disease incidence and severity throughout the world^7, 8^. The problem is accentuated by *R. solani’s* extremely broad host range and the high survival rate of sclerotia under various environmental conditions^9^.

There are no known rice varieties showing stable resistance to *R. solani* ^10–13^ and only partial resistance to ShB was detected by a survey of 6,000 rice cultivars from 40 countries in which no cultivar exhibiting a major gene for sheath blight resistance was identified^14^. To date, around 59 QTLs have been either mapped or evaluated for effectiveness against ShB disease under field conditions^11, 15–19^, and the potential of most published ShB-resistance QTLs in rice breeding is not clear.

In the absence of a desired level of resistance, the disease is currently managed by excessive application of chemical fungicides, which have drastic effects on the soil biota and are environmentally harmful. Artificial selection during rice domestication, has resulted in a significant reduction of genetic diversity in the gene pool among the present day rice cultivars^20^. This process has largely contributed to the genetic vulnerability of modern rice lines to various biotic and abiotic stresses. In this context, transgenic approaches offer new opportunities to broaden the genetic base by bringing novel genes/QTLs from genetically divergent rice lines into agronomically superior rice cultivars. To complement conventional breeding, genetic engineering is a promising strategy to generate crops with resistance against economically important plant diseases like rice ShB. Host-Delivered RNAi (HD-RNAi), also known as Host-induced gene silencing (HIGS) involves the production of double stranded RNA molecules targeting pathogen genes in the host where they are processed into short interfering RNA molecules (siRNAs). The siRNAs are taken by the pathogen upon infection inducing the RNA interference process and silencing the targeted gene in the pathogen^21^. HD-RNAi has been successful in engineering resistance against viruses^22^, insects^23^, nematodes^24^, parasitic plants^25^ and fungi^26^. The main advantage of HD-RNAi technology is that it can provide protection against pests and pathogens without introducing new proteins into the food and feed products. HD-RNAi has been successfully used against important fungal diseases such as barley powdery mildew fungus (*Blumeria graminis*)^27^, *Puccinia* genus^28^ and *Fusarium* ^29^.

In this study, host delivered RNAi (HD-RNAi) was used to produce rice varieties tolerant to the important pathogen, *R. solani*. Transgenic lines carrying a hybrid RNAi construct targeting two pathogen genes; pathogenicity MAP kinases *RPMK*1-1 and *RPMK*1-2 were produced. We observed increased ShB resistance in the transgenic lines accompanied by a concomitant decrease in fungal expression levels for the targeted genes, compared to controls.

## Materials and Methods

### Plant material and fungal strain

A japonica rice genotype, Taipei 309 (TP-309), susceptible to sheath blight disease was used for genetic transformation. A virulent isolate of *R. solani* anastomosis group AG-1 IA was obtained from the regional station Kapurthala (Punjab, India). This isolate was originally collected from the ShB rice fields and was maintained on potato dextrose agar (PDA) for further use. For production of fungal mycelia and sclerotia, *R. solani* was grown on PDA at 28 ± 1 °C under dark. The fungal mycelia were harvested five days after inoculation for phenotyping experiments.

### Identification of *Rhizoctonia solani* pathogenicity related gene

Present work was initiated in order to develop rice varieties resistant to *R. solani* using a novel genetic engineering approach called Host Delivered RNAi (HD-RNAi). However, as there is no report of pathogenicity related genes in *R. solani*, as an alternative approach, we first identified gene related to virulence in other necrotrophic fungi and later its homolog target gene was identified in *R. solani*. To start with, we selected MAP kinases genes related to signal transduction in host pathogen interaction. Exhaustive analysis was done using the Plant-Host interaction database (PHI-base) (http://www.phi-base.org/) and *R. solani* genome database (http://www.rsolani.org^30^) containing the first version of AG3. *R. solani* metagenome was then used to identify the homolog for these pathogenesis related (PR) genes. The gene products of these PHI-base hits were further analyzed using COBALT a multiple alignment tool to identify the genes that have been characterized to be crucial for virulence in several other fungal species. The selected target gene further was used as a query against *R. solani* genome database to identify two homologs for the candidate fungal *PMK*1 gene - *RPMK*1-1 and *RPMK*1-2 (Accession No. HQ003934.1 and HQ003936.1, respectively) and which were used for cloning of *R. solani PMK*1 gene.

### Quantitative Gene Expression Analysis of candidate PR gene

qRT-PCR was performed to study the expression analysis of both the homologs using specific primers (Table S1). For this leaves of control TP-309 plants were infected with *R. solani* mycelia and samples were collected at 12, 24 and 48 h post inoculation. Total RNA was isolated and cDNA was prepared from 3 µg of each of the total RNA samples using cDNA Synthesis Kit (Thermo Fisher Scientific Inc.). Relative expression of the genes was analysed and data obtained was normalized with the value of *Rhizoctonia solani* beta Tubulin primers and the relative fold change was calculated by using 2^−ΔΔC*t*^ method^31^. The thermal cycling conditions were initial DNA denaturation at 95 °C for 3 min followed by 45 cycles of amplification (denaturation at 95 °C for 10 s; primer annealing at 60 °C for 15 s and primer extension at 60 °C for 15 s).

### Construction of rice transformation vector

*R. solani* is a species complex consisting of genetically different AG groups. Genetic variation also exists between subgroups, within the same AG and between isolates from different regions. For our HD-RNAi approach to work, our RNAi constructs have to be AG specific and as AG1-1A *R. solani* is the primary causal agent for sheath blight in rice, cDNA of AG1-1A *R. solani* was prepared and from this, candidate genes were successfully cloned using degenerate primers. The RNAi cassette was produced into hairpin vector pSTARLING (http://www.pi.csiro.au/RNAi/vectors.htm); a vector specialized for high level constitutive RNAi expression in monocot plants. Two homologs of the selected target gene (*RPMK*1-1 & *RPMK*1-2) were used to prepare a hybrid RNAi construct using ~500 bp of each gene with the objective of silencing both the targets. The RNAi cassette was then cloned into a plant transformation vector to be used for genetic transformation of rice.

A modified rice transformation vector was constructed using pBlueScript SKII (Stratagene, USA) in which the hygromycin gene (*hpt*II) amplified from pCAMBIA 1305.1 was cloned at the *Sac*II restriction enzyme site in multiple cloning sites. In order to develop RNAi construct targeting the homologs of *RPMK* gene, the full-length ORF of 1086 bp was PCR amplified from the cDNA of *R. solani*, cloned and confirmed by DNA sequencing. The gene fragment was cloned in sense and anti sense orientation in the RNAi intermediate vector, pSTARLING-A at *Not*I site. Entire RNAi cassette (having promoter and terminator) was first released from pSTARLING vector by using *Not*I enzyme and then sub-cloned into pBS*hpt* vector again at *Not*I site and the construct was named as pBS*RPMK*. Details of the cloning steps are presented in Supplementary Fig. S1.

### Genetic transformation of Rice

For genetic transformation of rice, mature seeds were used as a starting material because they are readily available throughout the year and the bombarded tissues are contaminant free. Mature, dehusked seeds of Taipei (TP) −309 were surface sterilized and placed on callus induction medium containing MS medium^32^ supplemented with 3% sucrose, 0.8% (v/v) of agar-agar, 2 mg/l 2, 4-D, 500 mg/l proline and 400 mg/l casein hydrolysate. Seeds were placed in such a way that half of the embryo was in contact with the medium and the other half facing upwards. The cultures were incubated in dark at 25 ± 2 °C for callus induction. Embryogenic callus originating from scutellum was dissected from the seeds after 15 to 20 days of incubation and used as a target tissue for genetic transformation using a biolistic particle gun (PDS-1000/He, Biorad, Hercules, CA, USA). Around 25 calli derived from the individual seeds were placed at the centre of 90 mm diameter Petri dishes containing semisolid osmotic media (MS media with 0.8% bacteriological agar, 30 g/l mannitol and 30 g/l sorbitol) 4 h prior to bombardment and incubated at 25 °C. Preparation of gold particles was performed according to the standard protocol^33^. Gold particles coated with pBS*RPMK* plasmid DNA were then used for biolistic mode of genetic transformation of rice. Explants were bombarded with RNAi construct using the optimized conditions of 1100 psi helium pressure at 90 mm target distance using 1100 psi rupture disk pressure to produce stably transformed rice plants. In the experiment a constant vacuum of 25 mm Hg was used. Bombarded calli were kept overnight on the osmotic medium and then subcultured on to the callus induction medium added with 50 mg/l hygromycin for selection. Selection and regeneration of transgenic plants were done in the presence of hygromycin. During selection process, calli were subjected to three selection cycles of 15 days each on hygromycin media. Proliferating calli were than subcultured on to regeneration media (MS media containing 2.5 mg/l BAP + 1.25 mg/l NAA along with 50 mg/l hygromycin for selection). Transformation efficiency (%) was calculated as the number of hygromycin resistant callus events recovered per 100 embryogenic callus infected. Regenerated plantlets with good shoot and root growth were subjected to *in-vitro* hardening. For this, agar was removed from the roots and plantlets were kept in water for 5–7 days under culture conditions. Plantlets were then transferred to soilrite and covered with polythene bags. After one week plantlets were finally transplanted in soil and shifted to the National Phytotron Facility (NPF), IARI, New Delhi, India.

### Molecular characterization of transgenic plants

To determine the transgenic nature of plants, PCR was performed and for this genomic DNA was isolated from putative transgenic plants and wild type control plants using modified CTAB method^34^. All of the hygromycin resistant plants that survived on tissue culture selection media were analyzed by PCR using *RPMK* & *hptII* gene specific primers. The PCR was set in a 20 μl reaction volume containing 100 ng DNA, 10 pM each of primers, 10 mM dNTPs, Taq buffer (1×), 2.5 mM MgCl_2_ and 0.5U Taq DNA polymerase (Ferments, USA). The PCR profile used for *RPMK* gene targeting RNAi construct was 95 °C for 3 min followed by 35 cycles for 30 s at 94 °C, 1.5 min at 58 °C and 1 min at 72 °C for elongation and final extension at 72 °C for 10 min. For *hptII* primers, the PCR condition was 95 °C for 3 min followed by 35 cycles for 30 s at 94 °C, 1 min at 58 °C, 1 min at 72 °C for elongation and final extension at 72 °C for 10 min. PCR products were subsequently analyzed by electrophoresis on 1% (w/v) agarose gel. List of the primer sequence used for PCR are given in Supplementary Table S1.

Plants which were found positive in PCR analysis were further validated by Southern blot analysis^35^ to confirm the copy number. Due to absence of any *Eco*RI site within the cloned *RPMK* gene in recombinant pBS*RPMK* construct, this restriction enzyme was used in Southern blot analysis. Ten micrograms of genomic DNA from PCR confirmed transgenic plants and non transgenic control plants was digested using *Eco*RI (New England Biolabs) and size fractionated on a 1% TBE-agarose gel at 40 V. Following denaturation and neutralization, the DNA fragments were transferred to a Hybond-N nylon membrane (BIORAD) by capillary transfer in a neutral buffer. The membranes were UV cross linked at 1,200 J for 2 min. Southern blot hybridization was performed using DIG labelled probes following the standard protocol^35^. A 228 bp PCR product of the coding sequence of the *RPMK* gene (Supplementary Table S1) was labelled with DIG DNA labelling kit and used as a probe.

### Segregation analysis of transgene in T1 progenies

Segregation analysis was performed to check the inheritance pattern of *RPMK* in transgenic plants. Seeds collected from self crossed transgenic (T_0_) plants were screened for hygromycin resistance by germination test, in which T_0_ seeds were grown on hygromycin (50 mg/l) containing MS media and observation was recorded as hygromycin-resistant (HygR) and hygromycin-sensitive (HygS) T_1_ plants^36^. Non transgenic plants were used as control during the experiment. The goodness of fit of the observed segregation ratio for the transgene was tested against the Mendelian segregation ratio (3:1) using the chi-square (χ2) test. The χ2 values were calculated using the method given by Greenwood and Nikulin^37^.

### Functional characterization of transgenic plants for sheath blight resistance

The pure culture of *R. Solani* (Rs-K strain) was maintained in Petri dishes on Potato Dextrose Agar medium. Uniformed sized mycelia plugs were used for the preparation of the inoculums. Transgenic plants were evaluated by using two different ShB assay methods viz., “bioassay using detached leaves” and “bioassay using leaf sheaths intact or whole plant bioassay” for screening the transgenic lines, as described by Kumar *et al*.^38^. For the detached leaf assay, leaves from 8–10 weeks old transformants were surface sterilized and placed on the Petri plate over wet filter papers. The adaxial surface of the leaves was inoculated with 8-mm-diameter mycelial disc from the peripheral region of a 5-day-old PDA culture of *R. solani*. Petri plates were sealed for maintaining humidity and incubated at 28 °C. For each transformant, three biological replications were used and non-transgenic plants were used as control. Phenotypic observation was recorded at 0, 24, 48 and 72 hour post inoculation (hpi).

For whole plant bioassay, transgenic and control rice plants were inoculated in between the sheath at maximum tillering stage (45 days after transplanting) with *R. solani* by using mycelia plug along with a sclerotium. The inoculated sheath was wrapped with parafilm for better contact between the stem and fungus as well as for maintaining humidity. The water level (5–10 cm) was maintained constantly for ensuring enough humidity to promote disease development. Observation was recorded regarding lesion size and number of lesions after inoculation. All the experiments performed in the present MS have been repeated thrice and are reproducible. Highest Relative Lesion Height percentage (HRLH%) was calculated using the formula -

$${\rm{HRLH}} \% ={\rm{Length}}\,{\rm{of}}\,{\rm{the}}\,{\rm{highest}}\,{\rm{lesion}}\,({\rm{cm}})/\mathrm{Plant}\,{\rm{height}}\,({\rm{cm}})\times 100$$

### RNA isolation and real-time quantitative PCR analysis

For the isolation of total RNA, 100 mg of sheath blight infected plant samples (wild type control as well as transgenic) were used 96 hour after the inoculation. For this, sigma Spectrum Plant Total RNA kit was used and isolation was done following protocol provided by the manufacturer. Majority of the biomass in sheath blight infected rice samples is of rice origin and only a limited proportion is of fungus. Due to this, it is assumed that the total RNA which was isolated from infected rice tissues will have higher proportion of plant RNA as compared to pathogen i.e. *R. solani* RNA. Therefore, after giving DNase treatment 3 μg of total RNA from infected rice tissues were individually used for cDNA synthesis using cDNA Synthesis Kit (Thermo Fisher Scientific Inc.).

Real-time quantitative RT-PCR was performed using Power SYBR Green PCR Master Mix using Light Cycler 480 II PCR system (Roche) using manufacturer’s guidelines. The relative expression of *RPMK* gene present in RNAi cassette was analyzed by qRT-PCR using specific primers designed for each homolog (Supplementary Table S1). The thermal cycling conditions were initial denaturation of 95 °C for 3 min, 45 cycles of 95 °C for 15 s, annealing and extension at 60 °C for 15 s. Dissociation curve analysis was performed by fluorescence reading at 2° intervals between 60 and 95 °C to ensure that only one PCR product was amplified. All experiments were performed with three biological replicates and three technical replicates. Beta tubulin primer was used as internal control to normalize the data and the relative fold change was calculated by using 2^−ΔΔC*t*^ method^31^.

### Morphological characteristic of transgenic plants

The phenotypic performance of different transgenic rice lines at T1 generation growing under greenhouse conditions was evaluated with respect to the non-transgenic control. Different morphological parameters viz. plant height (cm), flag leaf length (cm), number of tillers and number of effective tillers was evaluated. The height of individual plants was measured as the distance from the soil surface to the tip of the flag leaf of the longest tiller. The flag leaf length was calculated as an average of all the flag leaves of an individual transgenic plant as well as non-transgenic line. Five randomly chosen plants from each transgenic line were evaluated for each parameter studied.

### Microscopic studies to study disease progression after inoculation

To study the disease progression in transgenic as well as wild type control rice, leaves originating from the main stem were harvested and challenged with *R. solani* using detached leaf assay. Leaves were placed over wet filter paper in the Petri plates and inoculated with mycelial disc of a 5-day-old PDA culture of *R. solani*. After infection leaves were fixed at 24 and 48 h time interval and then stained for observation of fungal development on the leaf surface. For fixation a modified method developed by Hein *et al*.^39^ was followed and leaves were fixed on filter paper soaked with 1:1 (v/v) Ethanol: Acetic acid for 24 h. After this, they were transferred on filter paper soaked with lactoglycerol (1:1:1 [v/v] lactic acid: glycerol: water). Finally samples were stained with aniline blue 0.1% (w/v) in 0.1 MK_3_PO_4_. Excess dye from the leaves was removed with 0.1M K_3_PO_4_. Leaves were mounted on to microscopic slides in 10% glycerol prior to microscopy. Observation of fungal structures as well as no. of infection cushions per microscopic fields in control as well as in transgenic rice leaves were recorded using compound microscope (Leica DM 750) and the pictures were taken using a coloured camera (Leica DFC 295).

## Results

### Identification of *Rhizoctonia solani’s* pathogenicity related MAP kinase genes

A search of the Plant-Host Interaction database (PHI-base) (http://www.phi-base.org/) revealed a number of gene families experimentally proven to be essential for pathogenicity in several necrotrophic fungal pathogens. Among them, MAP kinases were especially abundant with 25 pathogenicity related MAP kinase genes identified from 14 fungal species, indicating the essential role that signal transduction has in the disease establishment. Pathogenic MAP Kinase 1 (*PMK1*) was chosen for our study as it plays an important role in several pathogenic fungi including *Magnaporthe grisea*, *Colletotrichum lagenarium*, *Cochliobolus heterostrophus* and *Pyrenophora teres* ^40^. BLAST searches of the *R. solani* genome database identified two genes with a high level of homology to *M. grisea’s PMK*1; *RPMK*1-1 and *RPMK*1-2 (Accession No. HQ003934.1 and HQ003936.1, respectively) and primers were designed to clone both *RPMK* genes from *R. solani* by PCR (Supplementary Table S1).

### Expression Analysis of the *RPMK* genes

**Figure 1:**
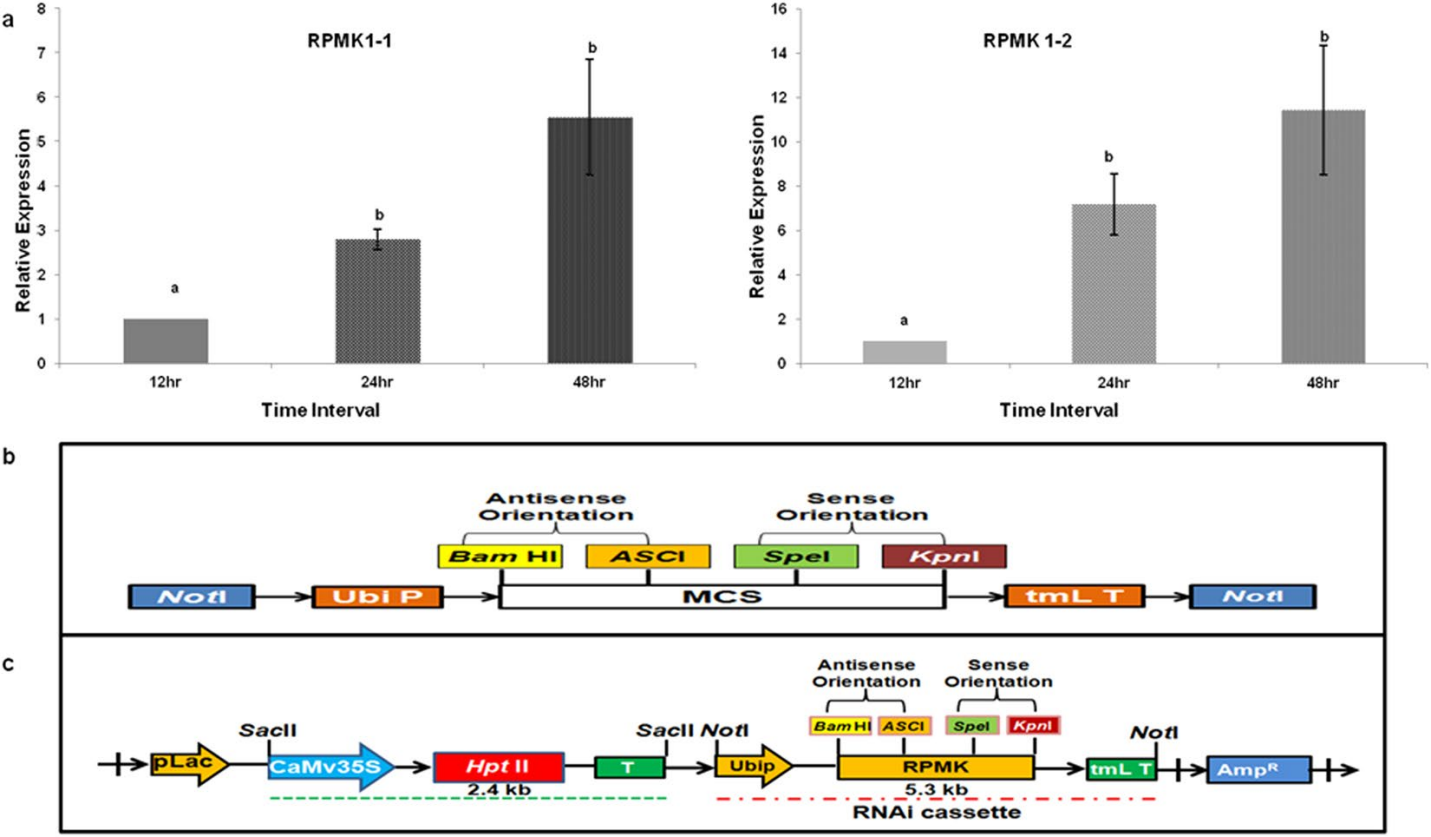
Expression analysis of *RPMK1-1* and *RPMK1-2* and schematic representation of HD-RNAi gene constructs. (**a**) Total RNA was isolated from plant tissue infected with *R. solani* 12, 24 and 48 hours post infection (hpi) and used for cDNA synthesis. Quantitative real time PCR was performed using gene-specific primers for *RPMK1-1* and *RPMK1-2*. *R. solani* beta tubulin levels were used for normalization purposes. For each gene, the relative mRNA levels at 12 hpi was given the arbitrary value of one and the remaining mRNA levels referred to it. Error bars indicate standard errors. (p = 0.05; ANOVA; with Tukey Kramer Multiple Comparison); (**b**) Partial map of the HD-RNAi construct where ~500 bp cDNA fragments for *RPMK1-1* and *RPMK1-2* were fused and cloned in sense and antisense orientation in the pSTARLING vector; (**c**) Sub-cloning of the RNAi cassette into a modified pBS vector containing the selectable marker gene *hpt*II.

Relative expression levels of *RPMK1-1* and *RPMK1-2* 12, 24 and 48 hours after inoculation of the susceptible rice cultivar TP-309 with *R. solani* were determined by qRT-PCR using *R. solani’s* tubulin as internal control for normalization purposes. Both *RPMK* genes were strongly induced during the initial infection process with *RPMK*1-1 showing 2.78 and 5.55 fold up-regulation and *RPMK*1-2 showing 7.20 and 11.43 fold up-regulation in infected rice leaves at 24 and 48 hpi, respectively (Fig. 1a). The strong upregulation of *RPMK*1-1 and *RPMK*1-2 suggests a possible role for these *R. solani* genes during the process of pathogenesis.

### Rice genetic transformation

*RPMK*1-1 and *RPKM*1-2 cDNA fragments were cloned by PCR from the AG1-1A *R. solani* isolate using specific primers (Supplementary Table S1). An RNAi cassette containing fused cDNA fragments for both homologs (*RPMK*1-1 and *RPMK*1-2) was cloned into the hairpin vector pSTARLING (Fig. 1b). The RNAi cassette was then cloned into a plant transformation vector containing the *hpt*II gene as selectable marker and the final HD-RNAi construct was named pBS*RPMK* (Fig. 1c).

**Figure 2:**
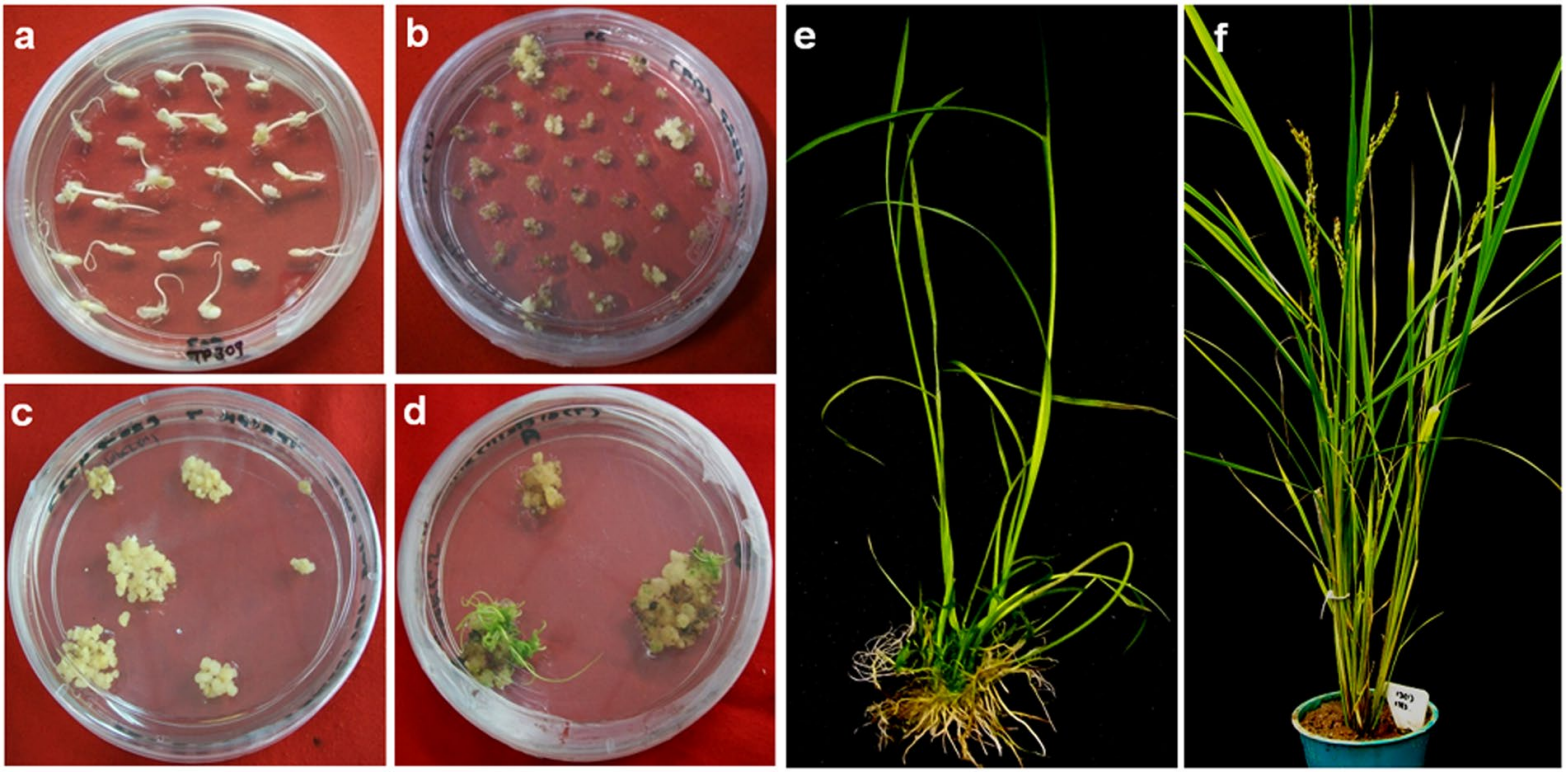
*In vitro* regeneration and stable genetic transformation of TP-309 rice plants. (**a**) Seeds grown on callus induction medium; (**b**,**c**) Transformed calli growing on selection medium; (**d**) Regenerating callus on selection medium; (**e**) Rooted putative transformants; (**f**) Putative transformant at panicle formation stage.

Transgenic rice lines containing the HD-RNAi construct were generated in the japonica variety TP-309 by bombardment of embryogenic callus (Fig. 2). In total, 20 putative T_0_ transgenic plants were produced from several transformation experiments with an overall transformation efficiency of 2.37% (Fig. 2a–f; Supplementary Table S2).

**Figure 3:**
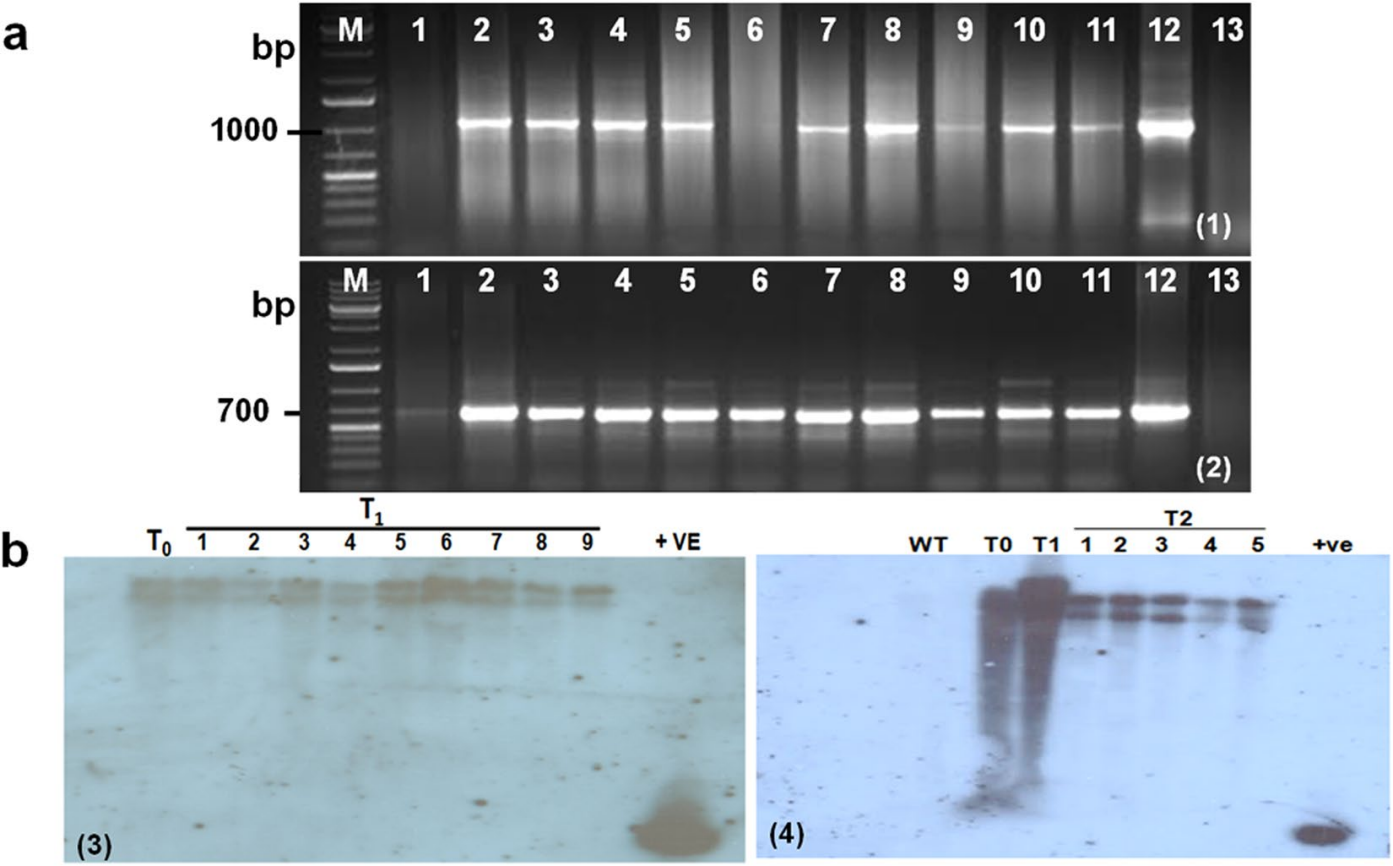
Molecular analysis of Transgenic TP-309 plants. (**a**) PCR analysis of putative transgenic plants using *RPMK* (1) and *hpt*II (2) gene specific primers. In both figures lane #12 is +ve control and lane #13 is −ve control; (**b**) Southern blot analysis showing the integration of the transgene in the T_1_ (3) and the T_2_ generation (4) of RP1 line. Original images of gel and Southern blots are also presented in supplementary information as Supplementary Fig. S2 and Supplementary Fig. S3.

Molecular analysis of putative transgenic rice plants from T_0_, T_1_ and T_2_ generations was performed by PCR and Southern blot hybridization to confirm the transgenic nature of the plants. PCR analysis confirmed the presence of 1086 bp and 670 bp amplicons from the *RPMK* and *hptII* genes, respectively in transgenic lines (named RP1-RP11), but no amplification bands were observed in the non-transgenic control plants (Figs 3a–1 and 2).

**Figure 4:**
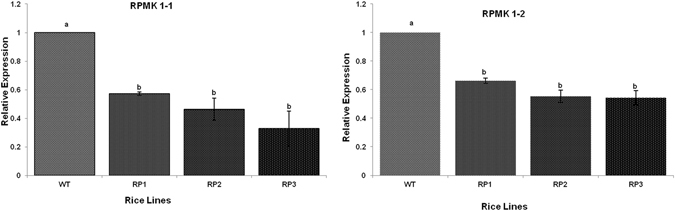
*RPMK*1-1 and *RPMK*1-2 expression levels in *R. solani* infecting control and HD-RNAi transgenic lines. Wild type and transgenic lines expressing the HD-RNAi constructs were infected with *R. solani*. Total RNA extracted from infected plant tissue 96 hpi was used for cDNA synthesis as a template for quantitative real time PCR with pathogen gene-specific primers. *R. solani* beta tubulin levels were used for normalization purposes. For each gene, the relative mRNA level measured in *R. solani* infecting non-transgenic controls was given the arbitrary value of one and the remaining mRNA levels referred to it. Error bars indicate standard errors (p = 0.05; ANOVA; with Tukey Kramer Multiple Comparison).

Three transgenic lines showed promising results on our preliminary phenotyping experiments (discussed below). Out of these three, T_1_ & T_2_ progeny of line RP1 which showed maximum resistance against sheath blight was analysed by southern hybridization. *Eco*RI digested genomic DNA of nine T1 and five T2 progeny of the RP1 event showed two bands, indicating the stable integration and inheritance of the HD-RNAi cassette (Figs 3b–3 and 4). No hybridization signal could be detected on DNA extracted from non-transgenic plants. The transgenic plants showed a normal phenotype and were fertile. Segregation analysis of the transgene in the T_1_ progeny of the transgenic lines revealed a HygR/HygS Mendelian segregation ratio of 3:1 for most lines suggesting a single integration event (Supplementary Table S3).

### Analysis of *RPMK*1-1 *& RPMK*1-2 expression levels in *R. solani* infecting HD-RNAi transgenic plants

To determine the effect of our HD-RNAi strategy on the targeted *R. solani* pathogenicity genes (*RPMK*1-1 *& RPMK*1-2) we measured the relative expression levels of these genes in fungi infecting transgenic lines and compared them with the levels present in fungi infecting wild type rice plants using qRT-PCR. Total RNA was isolated from infected plant tissue 96 h after the inoculation with the pathogen and cDNA synthesized by reverse transcription. Gene specific primers were designed for *RPMK1-1* and *RPMK1-2* outside the fragments used in the RNAi constructs to avoid possible artifacts. *R. solani’s* beta tubulin transcript levels were also measured and used for normalization purposes. Analysis of three independent transgenic lines showed a clear downregulation of *RPMK*1-1 and *RPMK*1-2 in *R. solani* infecting HD-RNAi Rice lines as compared to fungi infecting non-transgenic controls (Fig. 4). Silencing levels varied in all the three transgenic lines, although non-significant, with RP1showing a 57% reduction in expression levels for *RPMK*1-1 and 66% for *RPMK*1-2 (Fig. 4).

### Disease progression in HD-RNAi transgenic rice lines

**Figure 5:**
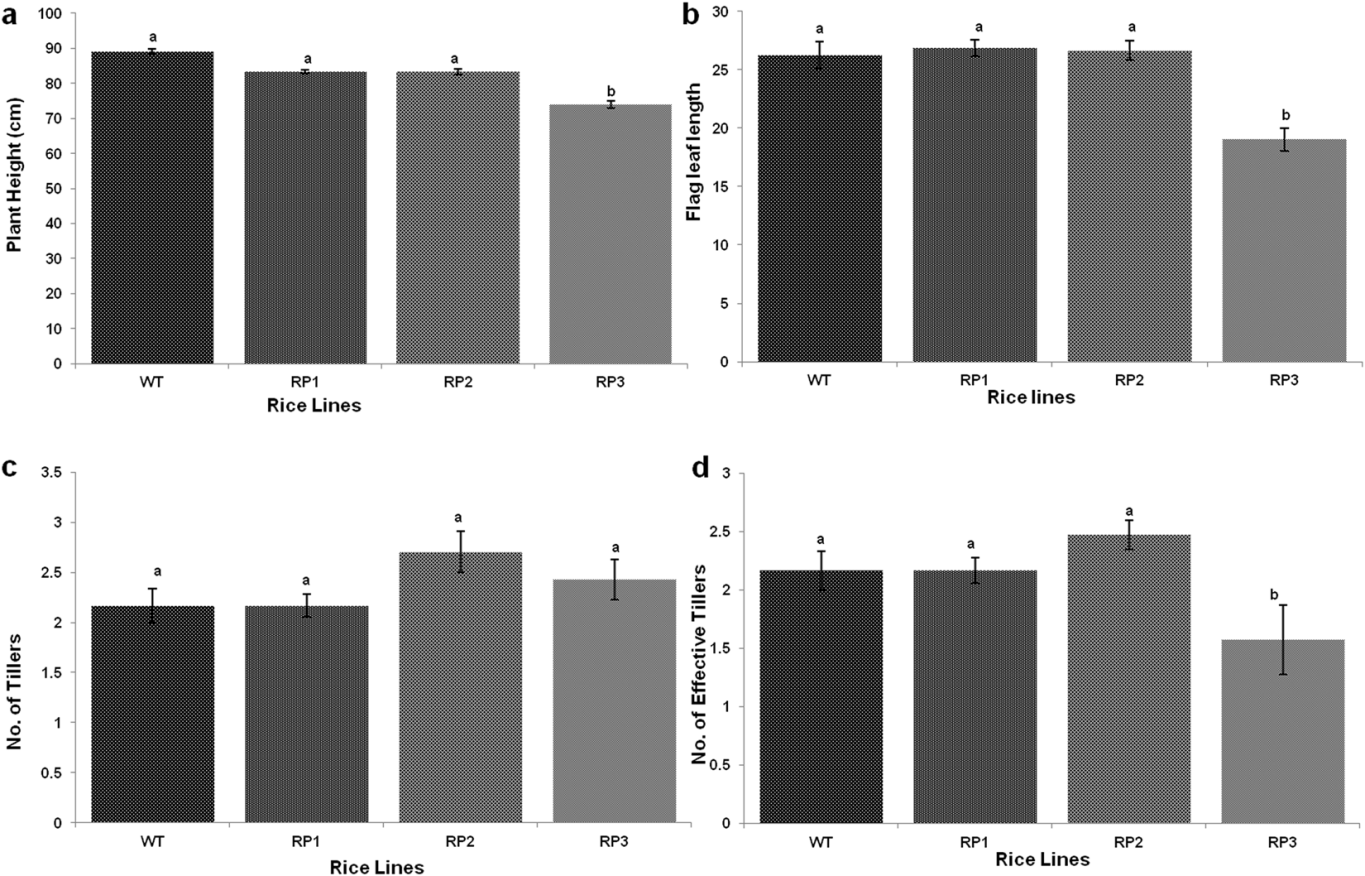
Morphological characterization of transgenic plants. (**a**) Plant height; (**b**) Flag leaf length; (**c**) Number of tillers; (**d**) Number of effective tillers Error bars indicate standard errors (p = 0.05; ANOVA; with Tukey Kramer Multiple Comparison).

No obvious morphological abnormalities were observed in the HD-RNAi transgenic lines (Fig. 5a–d). Although some developmental parameters showed slight variations between RP1 and WT plants, but these variations were statistically not significant.

**Figure 6:**
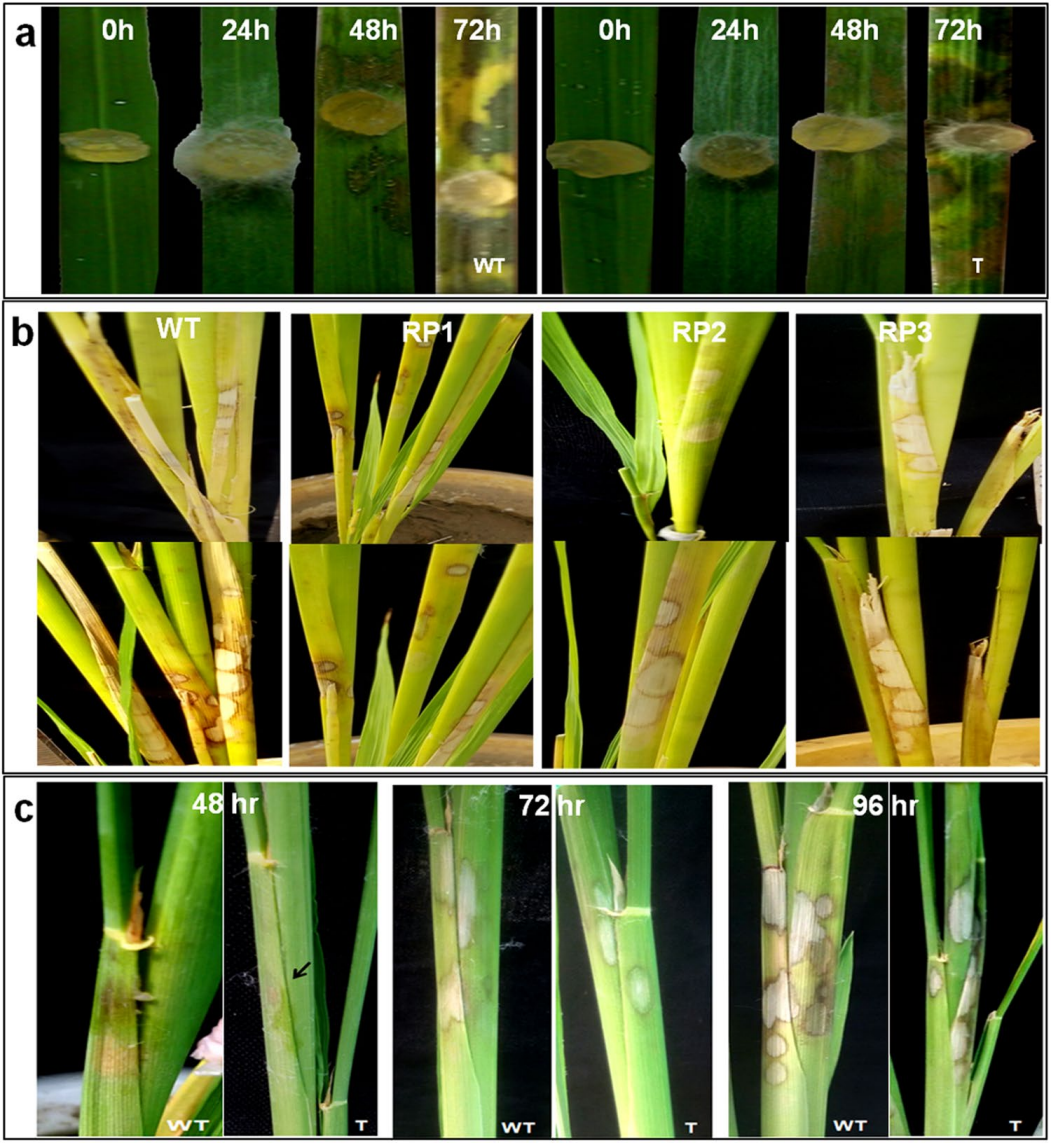
Transgenic plants with RNAi constructs exhibiting enhanced resistance against the sheath blight pathogen *Rhizoctonia solani*. (**a**) Detached leaf bioassays of transgenic lines and non-transgenic controls. (**b**) Whole plant of transgenic lines RP1, RP2 and RP3 and non-transgenic controls. (**c**) Whole plant bioassays of homozygous T2 plants from the RP1 line and non-transgenic controls.

**Figure 7:**
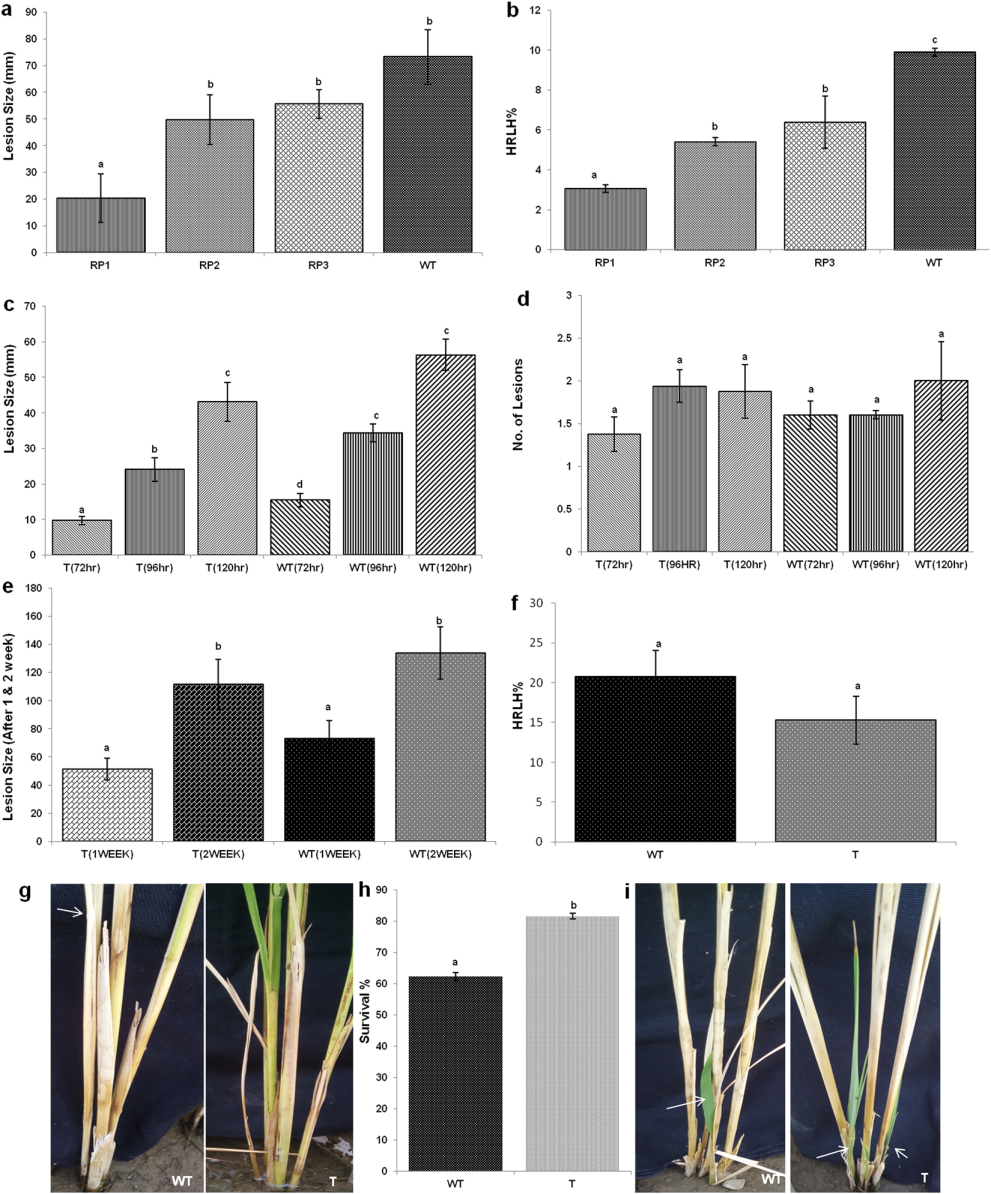
Disease progression and survival of transgenic and wild type control plants after *R. solani* infection. (**a**) Lesion size and (**b**) Highest Relative Lesion Height percentage (HRLH%) in T1 transgenic and non-transgenic controls. (**c**) Lesion size and (**d**) number of lesions in homozygous T2 plants from the RP1 transgenic line and non-transgenic controls at several times after infection. (**e**) Lesion size in homozygous T2 plants from the RP1 transgenic line and non-transgenic controls one and two weeks after inoculation. (**f**) HRLH% in homozygous T2 plants from the RP1 transgenic line and non-transgenic controls two weeks after inoculation. (**g**) Death of few tillers in wild type control as compared to transgenic plants after 2 weeks of infection; (**h**) Effect of *R. solani* infection on survival of tillers; (**i**) Emergence of new tillers was also reduced in wild type control as compared to transgenics after infection. Error bars indicate standard errors. (p = 0.05; ANOVA; with Tukey Kramer Multiple Comparison); WT = Non transgenic plants; T = Transgenic plants.

To evaluate the effectiveness of the HD-RNAi construct to confer resistance against *R. solani*, detached leaf assays were performed for the initial screening of the T1 transformants. These assays showed a reduced rate of lesion expansion in transgenic lines compared to non-transgenic controls (Fig. 6a). Additional experiments were performed using greenhouse acclimatized T1 transgenic plants grown in pots and inoculated with uniform mycelial plugs. Visual inspection of the inoculated lines showed a clear difference in disease severity between transgenic and non-transgenic lines (Fig. 6b). Quantitative measures of lesion size and Highest Relative Lesion Height (HRLH%) confirmed the visual observations showing a significant reduction in the whole plant bioassay for the transgenic lines (Fig. 7a,b). Combined, these results showed significant reductions in lesion size as well as delayed disease development in the transgenic lines compared to non-transgenic controls.

The RP1 line was chosen for further analysis and T_2_ homozygous transgenic plants subjected to whole plant bioassays. Disease symptom development was always less severe in the transgenic lines (Fig. 6c). Transgenic plants showed smaller lesion sizes in the initial stages of the disease (72 & 96 hpi) (Fig. 7c) and even though at 120 hpi the lesion size was comparatively smaller in transgenic plants compared to non-transgenic controls, this difference was not statistically significant. No statistically significant differences were observed between transgenic plants and non-transgenic controls in respect of lesion number (Fig. 7d). Further phenotypic analysis of lesion size was recorded one and two week post inoculation to study the disease progression and it was observed that visually transgenic plants appeared to perform better than non-transformed controls although these differences were not statistically significant (Fig. 7e). HRLH% two weeks after infection was lower in HD-RNAi transgenic lines compared to non-transgenic controls (15.29% vs 20.74%) (Fig. 7f). The lack of statistical support for the observed difference could be attributed to the total number of surviving tillers in transgenic and non-transgenic plants. It was observed that for some tillers most sheaths turned yellow within one week and by the end of second week the tiller completely died (Fig. 7g). As the data related to lesion size and HRLH% was taken only from the surviving tillers, this fact could distort the results and affect the statistical significance. In fact the percentage of surviving tillers in transgenic plants was significantly higher than in non-transgenic controls (Fig. 7h). We also observed that the emergence of new tillers in infected plants was higher in the transgenic plants (Fig. 7i).

### Microscopic observation of disease progression

**Figure 8:**
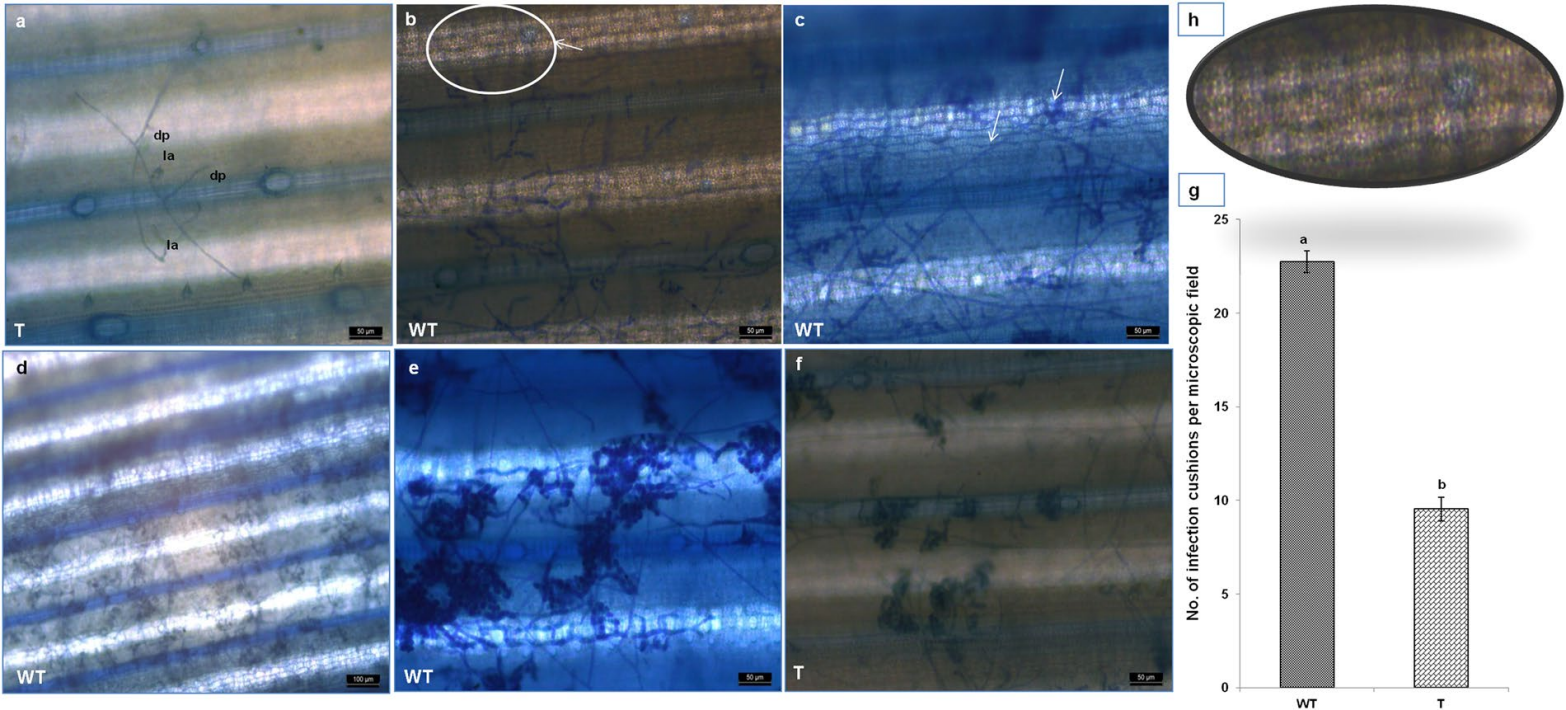
Disease development on transgenic HD-RNAi and control rice plants. (**a**) Hyphae developing side branches which either directly penetrate (dp) or develop into infection structures like lobate appressorium (la); (**b**) Hyphae showing zigzag pattern of growth inside the cells; (**c**) Hyphae invading host tissues via the epidermal cells and stomata; (**d**) Hyphae initiating infection cushion at the site of lesion 24 hpi in non-transgenic controls; (**e**) Densely formed Infection cushions leading to lesion development and necrosis of the cells in non-transgenic control (**f**) and transgenic HD-RNAi rice lines 48 hpi; (**g**) Number of infection cushions in transgenic plants and non-transgenic controls 48 hpi. (**h**) An enlarged section of panel b; WT = Non transgenic plants; T = Transgenic plants. Scale bar: 100 µm in panel (**d)** and 50 µm in rest of the panels.

Visual observations of infected leaves revealed that lesions started developing within 24 hpi with hyphae growing and colonizing the leaf surface within 48 hpi (Fig. 8). After colonizing the host surface fungal hyphae started developing side branching which either directly penetrate (dp) the cells or develop into infection structures like lobate appressorium (la) (Fig. 8a). Hyphae invaded the host tissue through the epidermal intercellular space as well as through stomata and trichomes and grew in a zigzag pattern once inside the leaf (Fig. 8b,c and h). We observed that in control plants hyphae tended to grow in bunches with several hyphae on the leaf surface while in transgenic plants hyphae were dispersed. The hyphae formed side branches and ultimately infection cushions. Control plants showed a larger hyphal mass and a higher number of appressoria subsequently leading to formation of infection cushions at 24 hpi (Fig. 8d), whereas in transgenic plants formation of infection cushions was not clear at this stage and hyphae showed only side branching and appressorium formation. Extensive colonization of fungal hyphae and appressorium was observed 48 hpi in control (Fig. 8e) and transgenic leaves (Fig. 8f) but control plants showed higher density leading to the formation of more prominent infection cushions. Lesions developed only underneath the infection cushions in both cases. Leaves of HD-RNAi transgenic plants showed significantly lower numbers of infection cushions (9.54/microscopic field) than non-transgenic controls (22.74/microscopic field) indicating an increased degree of resistance against *R. solani* (Fig. 8g). Additional microscopic observations showed that hyphal mat colonization was more extensive in non-transgenic control plants than in the transgenic lines.

## Discussion

Rice is staple food crop for a large portion of the world population, the majority of which lives in Asia. The effect that climate change will have on rice production is unknown and it could have an important impact on the ever increasing food demand to feed the growing world population. Plant pathogens are a real threat to worldwide agricultural production and fungal pathogens contribute significantly to yield loses^41^. *Rhizoctonia solani* Kuhn is a serious threat in rice growing areas as it has a wide host range and can infect more than 32 plant families and 188 genera^42^. The rice sheath blight pathogen infects at seedling, tillering and booting stages, usually starting on tissue near the water line in paddy fields with lesions spreading to the upper leaf sheaths and blades at later stages and subsequently killing the entire plant in severe cases. One of the conventional methods to combat plant fungal pathogens includes breeding for resistant lines, which is limited by the availability of resistance in the germplasm; this resistance can be overcome by evolution in fungal pathogenic races^43^. Many pathogenic fungi develop resistance mechanisms through genetic adjustment becoming less sensitive to agrochemicals^44^. This situation is very much true in the case of *Rhizoctonia* as there is a paucity of available host resistance in the available rice germplasm. Management of rice sheath blight is further complicated by the ability of the pathogen to survive in soil for a long time in the form of sclerotium and by its high genetic variability making resistance breeding difficult. Adding to these problems, the changing climatic conditions are enhancing the severity of rice sheath blight due to elevated CO_2_ atmospheric concentrations^45^. Novel and alternative strategies for the effective management of *R. solani* are urgently needed.

One promising approach is to target fungal pathogenicity genes using the HD-RNAi/HIGS approach. Transfer of dsRNA and/or siRNAs from plants to various pathogens has been demonstrated in a number of experimental systems^46^. Recent studies have confirmed the effectiveness of this approach to protect plants against pathogenic nematodes, insects, bacteria and fungi. A significant reduction in *Blumeria graminis* infection was observed in transgenic barley plants carrying a hairpin RNAi cassette targeting the fungal effector gene *Avra10* compared to non-transgenic control plants suggesting the trafficking of dsRNA or siRNA from host plants into pathogen leading to silencing of the fungal gene^27^. Virus-induced gene silencing (VIGS) which is similar to RNAi technology, was used to control infection of *Puccinia striiformis* and *Puccinia triticina* in wheat and it was shown that small RNA molecules can move from plant cells to fungal cells during the infection process and silence the target genes^28, 47, 48^.

In the present study, the HD-RNAi strategy was used to enhance resistance against the rice sheath blight pathogen *R. solani* in the susceptible rice variety TP-309. The strategy was conceptualized based on the fact that interactions between fungal pathogens and their host plants occurs via highly specialized cells called haustoria which are surrounded by the extra haustorial matrix bordered by plant and fungal membranes on either side, providing an interface for signal exchange as well as nutrient uptake^49^. This close contact also facilitates the uptake of dsRNA or siRNA molecules from the host plant cells by the fungal pathogens leading to RNAi-mediated silencing in the pathogen. Homology based gene silencing induced by transgene (co-suppression), antisense or dsRNA has been successfully demonstrated in plants against several pathogenic fungi, including *Cladosporium fulvum* ^50^, *Venturia inaequalis* ^51^, *Neurospora crassa* ^52^, *Aspergillus nidulans* ^53^, *Fusarium graminearum* ^54^, *Magnaporthe oryzae* ^55–57^ and *Fusarium oxysporum* ^29^. In this work we demonstrate that HD-RNAi is an efficient approach to control growth and development of the sheath blight pathogen *R. solani*. The success of this technology largely depends on the identification of suitable target gene/s in the infectious agent, but no pathogenicity related genes has been reported so far for *R. solani*. Comprehensive data-mining of the PHI database identified Pathogenic Map Kinase (PMK); Cyclic dependent protein Kinase A (CPKA) and G protein Beta subunit (GB) as promising targets for our study. Interestingly all three candidates seem to be involved in the same developmental pathway and are responsible for maturation of fungal appressorium, cuticle penetration and viability inside the host plant^58, 59^. *Magnaporthe grisea* a pathogen of economically important crops such as rice (*Oryza sativa*), barley (*Hordeum vulgare*), wheat (*Triticum aestivum*), and millet (*Panicum miliaceum*) has emerged as a model system for the study of plant-fungal interactions^60, 61^. During infection, the fungus produces specialized infection structures on the plant leaf surface called appressoria and through them it generates enormous turgor pressure to penetrate the underlying plant surface resulting in the development of lesions. Several signal transduction pathways regulating surface recognition, appressorium formation, and invasive growth in *M. grisea* have been identified^61, 62^. Out of three classes of MAPKs identified in ascomycetes as important for pathogenicity, the first class is represented in *Magnaporthe grisea* by *PMK1* (Pathogenicity MAP Kinase 1)^40, 63^. Xu and Hamer^40^ proved that MAP kinase and cAMP signalling regulate infection structure formation and pathogen growth and that *PMK*1 is required for appressorium formation and pathogenesis in *M. grisea* life cycle and it is essential for survival or growth of the fungus in rice plants. According to them *PMK*1 may act downstream of the cAMP signalling pathway and cooperative signalling between cAMP and a conserved MAP kinase pathway regulates key steps in fungal pathogenesis. *PMK*1 homologs have been characterized in other phytopathogenic fungi and proven to be essential for appressorium formation in all four appressorium-forming fungal pathogens examined to date: *M. grisea, Colletotrichum lagenarium, Cochliobolus heterostrophus*, and *Pyrenophora teres* with mutations resulting in pathogenicity loss and failure to colonize either healthy or wounded host tissues^64–66^. *PMK*1 homologs also play a major role in fungal pathogenicity in several filamentous fungi like *Botrytis cinerea, Gibberella zeae, Fusarium oxysporum* f sp. lycopersici, *Claviceps purpurea* and *Cochliobolus heterostrophus* ^67–70^ and they are required for fungal development and full virulence on hosts. All these studies concluded that the PMK1-mediated pathway may be conserved in many phytopathogenic fungi to regulate appressorium formation and other infection processes. On the basis of this evidence we identified two *PMK1* homologs in *R. solani* named, *RPMK*1-1 and *RPMK*1-2 and a hybrid RNAi construct was developed fusing a ~500 bp fragment from each cDNA with the objective of silencing both target genes. Expression of these genes during the process of pathogenesis has been confirmed by qRT-PCR analysis.

To determine whether our HD-RNAi approach affects the growth of *R. solani*, transgenic and non-transgenic control plants were challenged with the pathogen using detached leaf as well as intact plant. Both assays showed that the onset of disease was delayed in the transgenic plants compared to non-transgenic controls. We showed that *RPMK*1-1 and *RPMK*1-2 are strongly induced in *R. solani* upon infection of wild type rice plants, but in *R. solani* infecting transgenic HD-RNAi lines the induction is much less accentuated. Compared to non-transgenic controls, the level of down-regulation for *RPMK*1-1 and *RPMK*1-2 was on average 57% and 66%, respectively in homozygous T_2_ plants of the RP1 line (Fig. 4) and showed a positive correlation with an increased resistance against the pathogen (Fig. 6). We observed delayed progress of the disease in transgenic plants compared to non-transgenic controls leading to better survival rates in transgenic plants. The partial silencing observed for both target genes; *RPMK*1-1 and *RPMK*1-2 was not unexpected as RNAi usually results in gene down-regulation rather than complete silencing^71^. The increased survival rates observed in infected tillers of transgenic plants suggests that the levels of down-regulation achieved for *RPMK*1-1 and *RPMK*1-2 have an effect on the ability of the pathogen to colonize the plant. It was also observed that the emergence of new tillers from the base of the infected plants higher in transgenic plants suggesting a better ability to overcome the disease (Fig. 7i).

Our findings indicate that silencing of the *R. solani RPMK* genes is effective in inhibiting fungal growth by inhibition of mycelium formation during plant infection and highlights the potential of RNAi technology for effective control of fungal diseases, specifically where there is paucity of resistance in the natural germplasm. Sheath blight affects the sheath and laminar portions of the leaf but even though the infection process is the same in leaf lamina and sheath, there is a higher number of infection cushions on the leaf lamina compared with the sheath during early (24 hpi) infection^72^. Keeping this study in mind, detached leaves were inoculated with mycelial plugs of *R. solani* to observe disease progression in control and transgenic plants microscopically. After inoculation, *R. solani* hyphae started producing side branches which either proliferated or developed into one of two infection structures. One type of infection structure is a discrete aggregate of compacted hyphae which is called infection cushions. Another structure is lobate appressoria which are short, swollen branches forming lobes at the apex. Both structures are closely oppressed to the plant surface without apparent mucilaginous material^73^. After 24 hpi it was observed that hyphae formed side branches and branched several times with short swollen cells (lobate appressoria) in both transgenic and non-transgenic plants however, formation of side branches of fungal hyphae as well as formation of appressorium was much less abundant in transgenic plants. We observed that at the site of infection, infection cushions developed only in control plants at 24 hpi and lesions appeared underneath the infection cushions whereas the adjoining area showed formation of side branches in hyphae and appressoria. As transgenic leaves did not show any visible lesions at 24 hpi they also lacked infection cushions. Plant resistance against fungal pathogens relies on inhibiting the development of infection structures such as infection cushions or lobate appressoria and disease severity correlates with the number and size of infection structures^74^. Infection cushions play an important role in disease progression as they cause enzymatic degradation of the leaf surface allowing physical penetration of hyphae with resistant cultivars overcoming pathogens by their cuticular wax on the outer sheath surface^5^. Our observations that transgenic leaves show lower number and reduced size of infection cushions 48 hpi support a possible role for *RPMK*1-1 and *RPMK*1-2 in appressorium formation and overall viability of the fungus inside the host. The whole plant bioassays confirmed the detached leaf assays.

Our results prove that it is possible to down-regulate *R. solani* pathogenicity genes using HD-RNAi. The level of silencing achieved was sufficient to delay disease progression and increase the rate of survival. The transgenic rice lines with increased sheath blight resistance can provide economic and environmental advantages to farmers over existing varieties.

## Electronic supplementary material

Supplementary Information
