# Supplementary material for: Host Delivered RNAi, an efficient approach to increase rice resistance to sheath blight pathogen (Rhizoctonia solani)
Source: Sci Rep. 2017 Aug 8;7:7521. doi: 10.1038/s41598-017-07749-w (PMC5548729; doi:10.1038/s41598-017-07749-w)
Supplement: Supplementary file 1 — Supplementary Information [file 41598_2017_7749_MOESM1_ESM.pdf]

Host Delivered RNAi, an efficient approach to increase rice resistance to sheath blight pathogen (*Rhizoctonia solani*)

Ila Mukul Tiwari[1], Arun Jesuraj[2], Richa Kamboj[1], B.N. Devanna[1], Jose R Botella [2], TR Sharma[1,3*]

1 National Research Centre on Plant Biotechnology, Pusa Campus, New Delhi-110012, India

2 School of Agriculture and Food Sciences, The University of Queensland, Brisbane, QLD, Australia

3 Present address- National Agri-Food Biotechnology Institute (NABI), Mohali, Punjab, India -160071

* T.R. Sharma

trsharma1965@gmail.com , trsharma@nabi.res.in

Supplementary Table S1: List of primers used in Genetic Transformation of Rice using HD-RNAi approach

**For qRT-PCR**	
RPMK1-1 F	5′ CTCAAGTTCTTGTCCGAGGCTGGTATTATTTCTA 3′
RPMK1-1 R	5′ TGGGCATGATCGTCGCTAAGGTCCTGC 3′
RPMK1-2 F	5′ TCACTGGACGACTTTTATGCTATCAACTCG 3′
RPMK1-2 R	5′ GTTGAGGGTTAAAAGTTAGGCATTTCTCCATG 3′
BTUB F	5′ ATTGGTAACTCGACTGCTATCC 3′
BTUB R	5′ GTGAACTCCATCTCGTCCATAC 3′
**Molecular analysis of Rice Transformants**	
RPMK F	5′ TATTTTATCTACCAAACGCTTCG 3′
RPMK R	5′ ATCTCGTTGCAACGTATTCCGTC 3′
*hpt*II F	5′ TCAACACATGAGCGAAACCC 3′
*hpt*II F	5′ AACTGTGATGGACGACACCG 3′
**For Southern Hybridication**	
RPMK SouF	5′ CGTCTGGTCCGTTGGTTGTA 3′
RPMK Sou R	5′ GAATGTAATCGCGGGATCGT 3′

Supplementary Table S2: Recovery of transgenic rice plants derived from the scutellar calli transformed with a RNAi construct (of *R. solani* pathogenesity gene RPMK) using Ballistic approach

Description	Experiment 1	Experiment 2	Experiment 3	Experiment 4
**Number of Calli placed on selection medium**	119	240	321	175
**Hygromycin resistant calli after 3rd selection cycle**	18	33	45	22
**No. of PCR positive Transgenic events**	3	6	7	4
**Transformation efficiency (%)**	2.53	2.5	2.18	2.28

Supplementary Table S3: Segregation analysis of T_1 progeny of rice transgenic lines with *RPMK* gene

**Transgenic event**	**Total no. of T_1 seeds**	**Hygromycin**		**χ^2**	
		**Resistant**	**Susceptible**	**Ratio**	**value**
WT	50	-	50	-	-
RPMK RP1	30	24	6	3:1	0.4
RPMK RP2	30	24	6	3:1	0.4
RPMK RP3	44	25	19	-	7.76

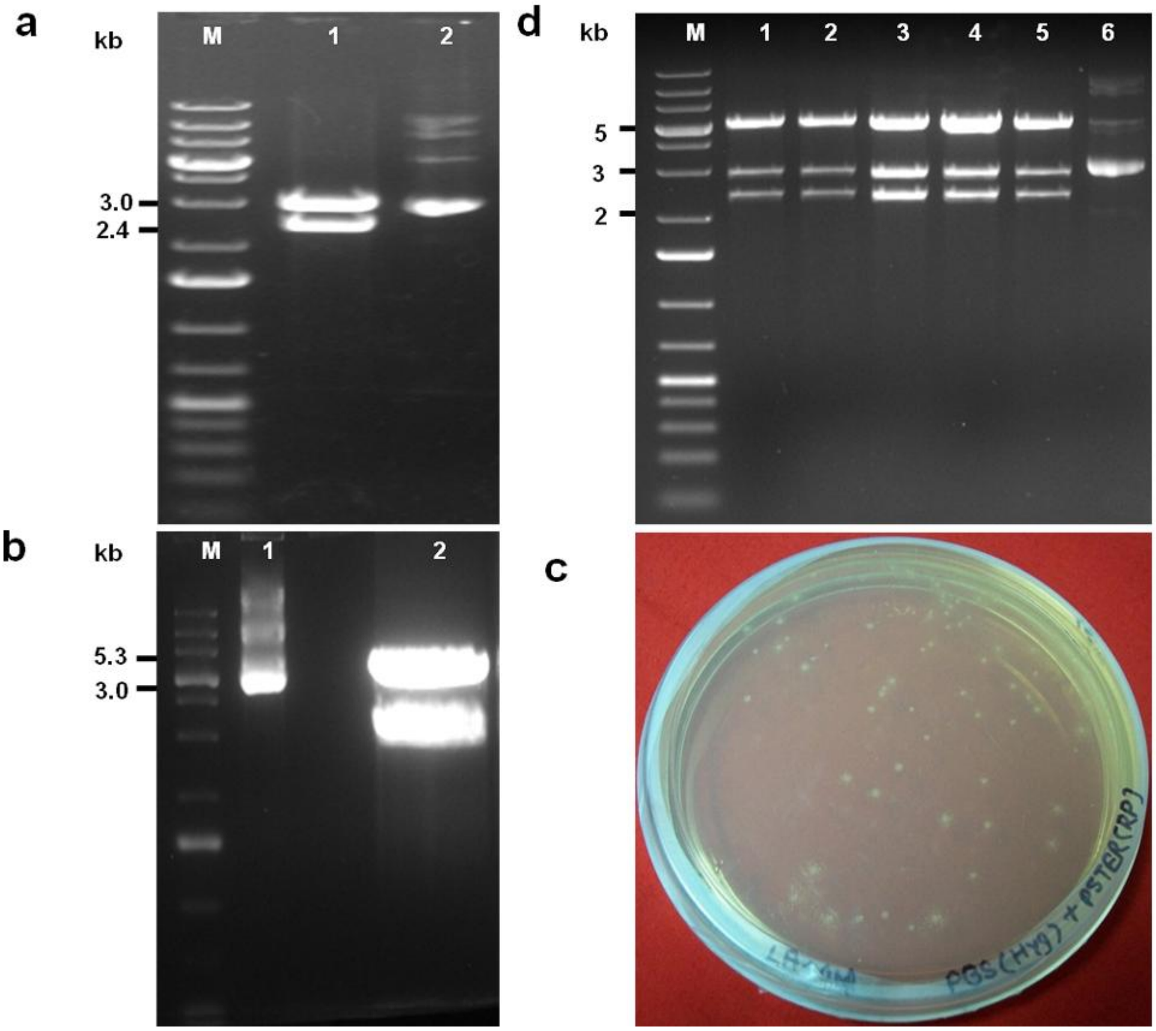

Supplementary Fig S1: Cloning of RPMK gene and confirmation of recombinant clones. (a) Confirmation by restriction digestion using *Sac*II restriction enzymes shows release of *hpt*II gene fragment of 2.4 kb and *pBS* vector backbone of 3 kb; (b) Release of RNAi cassette from pSTARLING vector by *Not*I digestion; (c) Recombinant clones with modified pBS vector + RNAi cassette growing on a selection plate; (d) Confirmation of recombinant clones by restriction digestion with *Not*I and *Sac*II - releasing RNAi cassette of 5.3kb, *hpt*II gene of 2.4 kb and *pBS* vector backbone of 3kb.

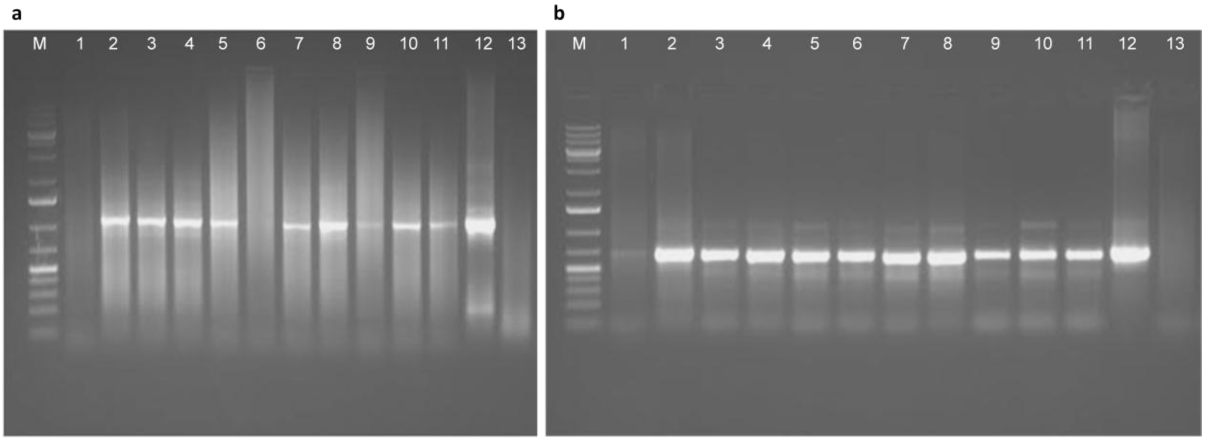

Supplementary Fig S2: Molecular analysis of Transgenic TP-309 plants: PCR analysis of putative transgenic plants using (a) RPMK gene specific primers and (b) *hpt* gene specific primers. In the above figures lane no 12 is +ve control and Lane no 13 is –ve control

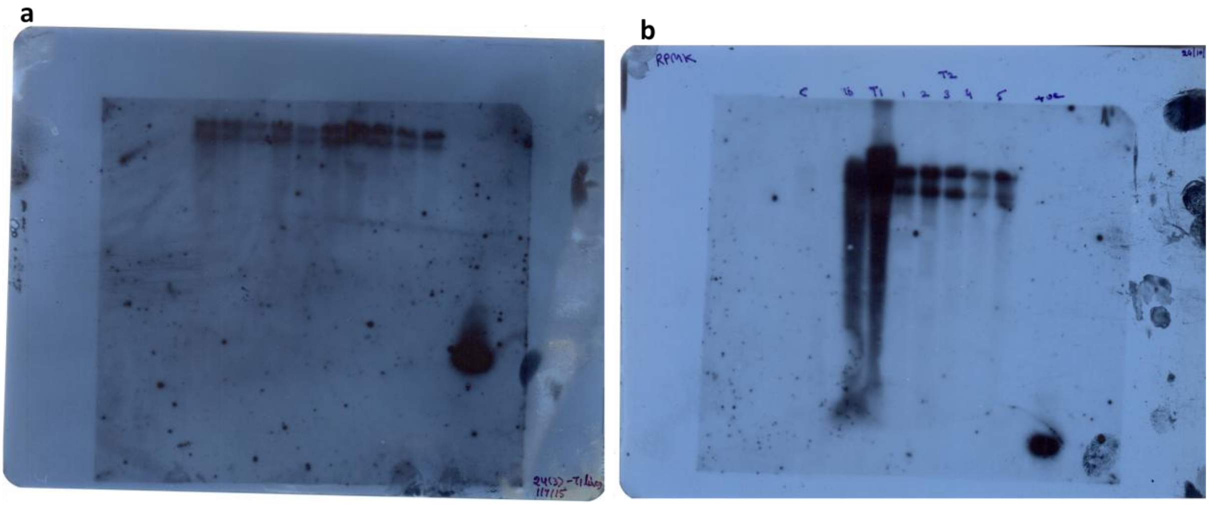

Supplementary Fig S3: Molecular analysis of Transgenic TP-309 plants. Southern blot analysis showing the integration of the transgene in the line RP1 (a) In T1 generation and (b) T2 generation.
